# Spatial optimization of transthoracic diffuse optics for myocardial ischemia in mice: a Monte Carlo study

**DOI:** 10.1117/1.JBO.31.10.105001

**Published:** 2026-10-14

**Authors:** Tao Yang, Guangqi Chen, Danni Wei, Yongle Wang, Sifan Chen, Jiayu Hu, Le Pan, Henggui Zhang, Li Wang, Jin Li, Weichao Liu

**Affiliations:** a Southwest Medical University, Key Laboratory of Medical Electrophysiology, Ministry of Education & Medical Electrophysiological Key Laboratory of Sichuan Province, Institute of Cardiovascular Research, Luzhou, China; b Southwest Medical University, Medicine & Engineering & Informatics Fusion and Transformation Key Laboratory of Luzhou City, School of Medical Information and Engineering, Luzhou, China; c Nuclear Medicine and Theranostics Key Laboratory of Sichuan Province, The Affiliated Hospital of Southwest Medical University, Department of Nuclear Medicine, Luzhou, China

**Keywords:** diffuse optics, myocardial ischemia, heart, Monte Carlo, near-infrared spectroscopy

## Abstract

**Significance:**

Continuous, noninvasive assessment of myocardial ischemia in intact mouse models poses a significant technical challenge. Conventional diffuse optical techniques are mostly limited to *ex vivo* hearts or open-chest animal models due to severe signal attenuation and tissue interference caused by the complex thoracic anatomy. Empirical placement of photoelectric probes over the cardiothorax further undermines the sensitivity and specificity of cardiac signal detection.

**Aim:**

This study aims to systematically characterize photon transport dynamics within the mouse thorax, determine the optimal light Source–Detector (S-D) spatial configuration, and quantitatively evaluate the theoretical feasibility of transthoracic diffuse optics for detecting varying degrees of myocardial ischemia.

**Approach:**

Using a high-fidelity 3D voxelized model derived from realistic mouse anatomical data, we performed Monte Carlo photon transport simulations at 800 nm. A high-density virtual array of sources and detectors was deployed on the body surface to determine the optimal S-D positions for cardiac signal extraction. Myocardial ischemia was simulated by modulating the optical coefficients of myocardial tissue, enabling the investigation of the relationship between surface-captured optical signals and ischemia severity.

**Results:**

The simulations identified an optimized S-D configuration that maximizes the detection sensitivity for deep myocardial signals while minimizing interference from overlying tissues. Notably, under this configuration, the diffuse optical signals collected at the body surface showed a strong linear correlation with the degree of myocardial ischemia.

**Conclusions:**

An optimized S-D configuration was demonstrated for establishing a linear correlation between optical signal variations and ischemic severity. This study provides a theoretical framework toward diffuse optics for *in vivo* assessment of cardiac hemodynamics and tissue oxygenation in murine models.

## 1 Introduction

Acute myocardial ischemia (AMI) and its subsequent heart failure represent a leading global health threat.^1^ In cardiovascular disease research, especially for mechanistic studies and drug development, mice are the most critical and widely used animal models. However, conventional methods for assessing murine cardiac function, such as electrocardiography and echocardiography,^2^^,^^3^ often fail to provide continuous, regional information about tissue microcirculation and hemodynamics.

In recent years, diffuse optical techniques have shown great potential for oxygenation monitoring in deep tissues (e.g., brain and skeletal muscle) due to their noninvasive nature, ability for continuous monitoring, and high temporal resolution. Previous studies using time-resolved near-infrared spectroscopy (NIRS) on isolated hearts showed that left coronary artery occlusion led to a significant increase in tissue deoxygenation signals, while oxygenation signals rebounded significantly after cardiopulmonary resuscitation.^4^ Gussakovsky et al. utilized NIRS to quantify parameters such as hemoglobin concentration and tissue oxygen saturation in isolated perfused porcine hearts,^5^ and applied it to monitor myocardial oxygen balance during open-chest surgeries.^6^ Similarly, laser speckle imaging has been employed to evaluate coronary blood flow dynamics following myocardial ischemia-reperfusion injury in both adult and aged mice.^7^ Despite these promising advancements, existing diffuse optical techniques remain predominantly restricted to *ex vivo* isolated hearts or require highly invasive open-chest procedures. Therefore, there is an urgent, unmet need for a noninvasive, transthoracic diffuse optical strategy capable of continuously evaluating cardiac oxygenation function in intact rodent models.

The cardiothoracic anatomy is characterized by complex, three-dimensional heterogeneous structures, encompassing highly absorbing mammalian skeletal muscles^8^ and highly scattering lung tissues. In particular, the profound photon absorption by the chest wall musculature severely attenuates the optical signals that manage to penetrate to the deep human myocardium and diffuse back to the tissue surface.^9^^,^^10^ Lacking a priori knowledge regarding deep photon migration pathways, the placement of sources and detectors in our previous *in vivo* NIRS experiments for rat cardiac monitoring relied heavily on empirical trial and error.^11^ Such suboptimal probe configurations are not only highly time-consuming but also prone to severe signal contamination, where the acquired optical signals predominantly originate from overlying superficial tissues rather than the targeted myocardium. Consequently, this confounding effect significantly compromises both the sensitivity and specificity of ischemia detection. Therefore, a systematic approach is urgently required to quantitatively characterize photon migration dynamics within the complex cardiothoracic anatomy, thereby providing a robust scientific foundation for designing the optimal source–detector (S-D) configuration.

To address the aforementioned spatial optimization challenge, Monte Carlo (MC) photon transport simulations, integrated with high-fidelity anatomical voxel models, have emerged as the "gold standard".^12^^,^^13^ By simulating the stochastic random walks and attenuation of a vast ensemble of photons within complex heterogeneous tissues—such as the chest muscle, ribs, lungs, and the heart—this method can precisely delineate the spatial distributions of photon absorption, scattering, and sensitivity. Consequently, such simulations provide crucial theoretical insights for optimizing the S-D configuration. Furthermore, it is well-established that acute ischemia induces structural alterations in cardiomyocytes,^14^^,^^15^ leading to measurable changes in tissue optical properties, particularly the scattering coefficient. Crucially, these optical parameters can be precisely adjusted within the simulation model to mimic different degrees of ischemia. This framework thus enables the quantitative clarification of the intrinsic relationship between surface-detected optical signals and the severity of myocardial ischemia, ultimately establishing the theoretical feasibility and validity of this non-invasive monitoring technique.

In our previous work that utilized Monte Carlo simulations based on realistic human cardiothoracic imaging datasets, we demonstrated that near-infrared photons can successfully penetrate the chest wall, which enables the extraction of deep myocardial optical signals.^9^^,^^10^ However, the application of diffuse optical technologies for cardiac monitoring in murine models, particularly for the non-invasive assessment of myocardial ischemia, remains a significant theoretical and methodological gap. This study aims to construct a high-fidelity MC photon transport simulation model based on a realistic cardiothoracic dataset to systematically determine the optimal S-D spatial configuration for the mouse model. Furthermore, our ischemia simulations reveal, for the first time, a significant linear correlation between the surface-captured diffuse optical signals and the severity of myocardial ischemia under the optimized S-D layout. Ultimately, this work provides essential theoretical support for the future hardware development and *in vivo* translation of multichannel small-animal cardiac diffuse optical systems.

## 2 Methods and Materials

### 2.1 Digimouse Atlas and the Thoracic Voxel Model

The thoracic voxel model was constructed from Digimouse, which was generated using coregistered CT and cryosection images of a normal male mouse with a matrix size of 380×992×208 and 0.1 mm cubical voxels.^16^ Digimouse is a well‑established 3D whole‑body mouse atlas, from which are segmented the whole brain, heart, lungs, liver, stomach, skeleton, skin, and 22 other tissues. A surface rendering of the 3D mouse atlas from Digimouse is presented in Fig. 1(a). The original voxel matrix with dimensions of 380×992×208  voxels was resized into a new matrix with 208×308×992  voxels. To reduce the computational complexity, a thoracic voxel model with dimensions 200×300×230 was extracted from [9, 51, 291] to [208, 350, 520], as shown in Fig. 1(b).

**Fig. 1:**
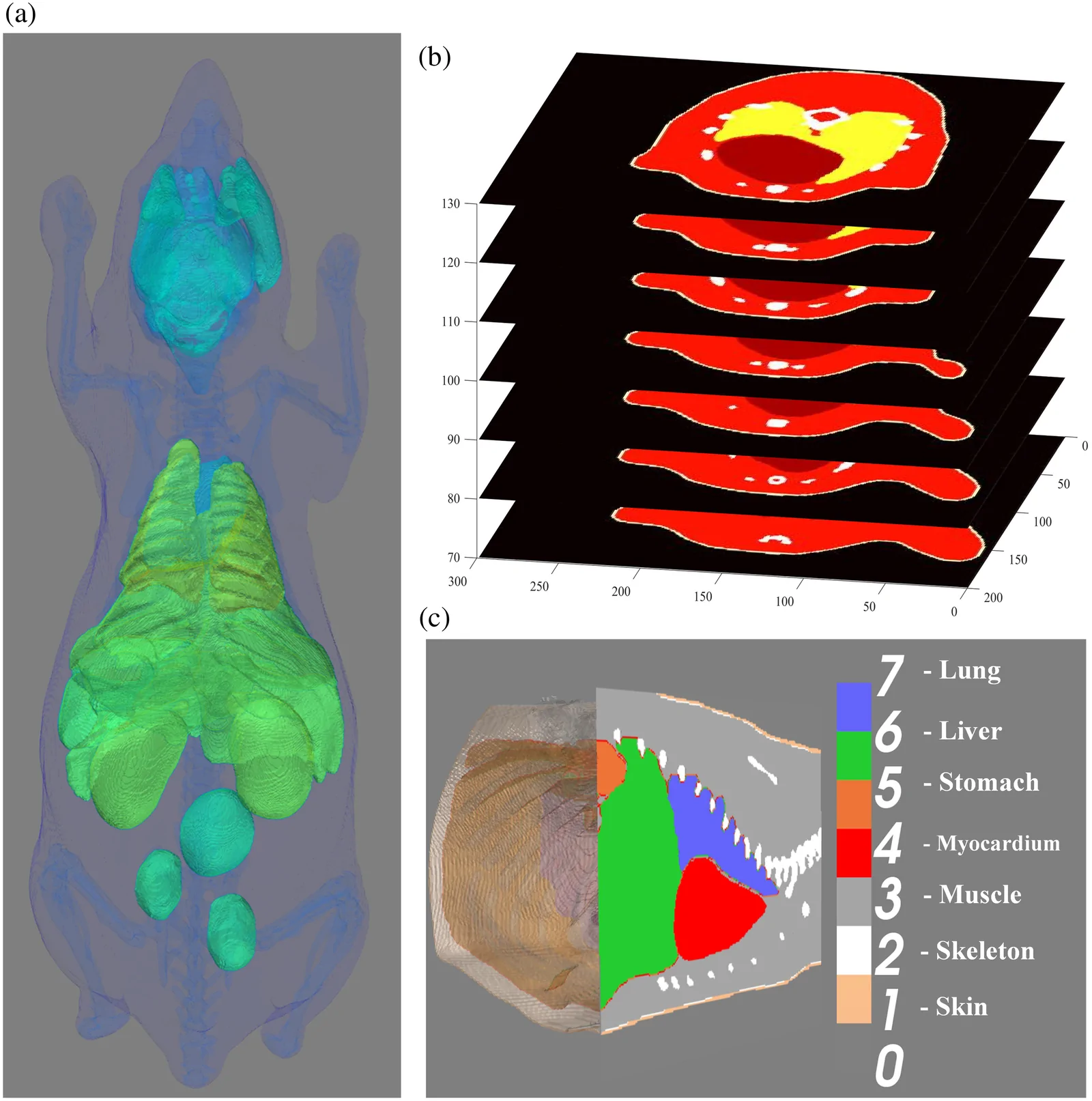
(a) Surface rendering of the Digimouse atlas. (b) Labeled thoracic tissue model with 200×300×230  voxels extracted from the Digimouse atlas. (c) Tissue segmentation of the Digimouse images: skin, skeleton, muscle, myocardium, stomach, liver, and lung, labeled as 1 to 7, respectively.

A limitation of the Digimouse atlas is that it collectively classifies muscle and skin tissues as “skin”. To make the simulation consistent with real-world conditions, we employed image edge detection and erosion algorithms, where the outermost two voxel layers were characterized as skin, and the remaining inner skin voxels were categorized as muscle. The thickness of mice skin is about 200  μm, and the two-layer cubical voxel is just 0.2 mm.^17^ As rendered in Fig. 1(c), the new thoracic voxel model includes skin, skeleton, muscle, myocardium, stomach, liver, and lung.

### 2.2 Parallel Monte-Carlo Simulation of Model

In a previous study, our group proposed a parallel GPU Monte Carlo model on 3D voxelized media (cuMCVM), 32.6 times higher than the nonGPU model in a human head simulation.^18^ cuMCVM is a parallel computing upgraded version of MCVM.^19^ In this study, the cuMCVM framework was employed for light propagation simulation in the mice heart tissue. In this study, the workflow for cardiac photon transport simulation using this framework is outlined as follows:

1. Generate a thoracic voxel model with dimensions of 200×300×230 from Digimouse, comprising seven labeled tissues (1 to 7): skin, skeleton, muscle, myocardium, stomach, liver, and lung.
2. Prepare the MCVM/cuMCVM simulation profile, which a total of $10^{7}$ input photons, the position and direction of the light source, the voxel model configuration, and the optical coefficients. The absorption coefficient, scattering coefficient, anisotropy, and refractive index are detailed in Table 1. The running time of a single simulation is about 30 min under the cuMCVM framework.
3. Quantify optical diffusion. Following the simulation, light absorption, light fluence distribution, and output photon counts are extracted. Furthermore, two pathlength factors are calculated: the differential pathlength factor (DPF) and the partial pathlength factor (PPF). DPF is the scaling factor that relates S-D separations to the average path length that light travels.^20^ In contrast, PPF refers to a similar scaling factor, but one that assumes a focal change. A PPF therefore represents a proportionality factor converting the S-D separation into the average path length that light travels through a change of optical properties. In this work, we compute two core metrics based on this framework: the PPF for myocardial tissue (${\mathrm{PPF}}_{\mathrm{myocard}}$), which indicates the sensitivity of the detected light intensity signal to optical property changes in the myocardium, and the ratio of ${\mathrm{PPF}}_{\mathrm{myocard}}$ to DPF, which quantifies the relative contribution of myocardial tissue to the overall detected signal.^10^

**Table 1:** Optical properties of thoracic tissues at 800 nm.

Label	Tissue type	$\mu_{a}$ (${\mathrm{cm}}^{-1}$)	$\mu_{s}$ (${\mathrm{cm}}^{-1}$)	g	n	References
1	Skin	0.20	73.7	0.715	1.37	21, 22
2	Skeleton	0.11	291	0.936	1.43	23
3	Muscle	0.54	66.7	0.93	1.40	24
4	Myocardium	0.92	103	0.93	1.4	25, 26
5	Stomach	0.78	47.0	0.761	1.4	27
6	Liver	0.80	56.6	0.929	1.4	28
7	Lung	1.00	95.0	0.91	1.4	29

### 2.3 Optical Absorption and Scattering During Myocardial I/R at 800 nm

Based on the validated absorbance spectra of hemoglobin previously established by Steve Jacques^30^ and Scott Prahl,^31^ the absorption spectra of HbR and ${\mathrm{HbO}}_{2}$ intersect at the isosbestic point at approximately 800 nm. Consequently, we assumed that myocardial light absorption remains constant during ischemia at this wavelength. Conversely, scattering coefficients decrease significantly during severe infarction occlusion.^15^^,^^32^ Therefore, the severity of myocardial ischemia can be represented by the ratio of the scattering coefficient of ischemic myocardium ($\mu_{s}$) to that of normal tissue ($\mu_{s0}$). A lower ratio $\mu_{s}/\mu_{s0}$ indicates more severe ischemia.

During myocardial ischemia (non-occlusion), blood flow through the tissue slows down, whereas intramyocardial blood volume or total hemoglobin content remains relatively stable. We therefore assume isovolumetric absorption in the myocardium at 800 nm during ischemia. In contrast, the tissue scattering coefficient decreases markedly upon the onset of myocardial ischemia. In this simulation study, we investigate the relationship between the severity of myocardial ischemia and changes in the scattering coefficient.

## 3 Results

### 3.1 Center of the Heart and Incident direction

Directing light from the skin toward the center of the heart ensures that the maximum number of photons reaches the myocardium. To optimize the diffusion of photons throughout the heart, it is critical to accurately determine both the heart’s center and the direction of the incident light. We defined the center of the heart as the endpoint of the incident light vector. The number of heart voxels in each slice was calculated across the X–Y, X–Z, and Y–Z planes to pinpoint this center, with the results shown in Fig. 2(a). As illustrated in Figs. 2(b)–2(d), the largest numbers of heart voxels were observed at the 135th, 144th, and 120th slices on the Y-axis (X–Z plane), Z-axis (X–Y plane) and X-axis (X–Z plane), respectively. The intersection of the three planes, at the coordinate [120, 144, 135], was established as the heart center and the endpoint for the incident light vector.

**Fig. 2:**
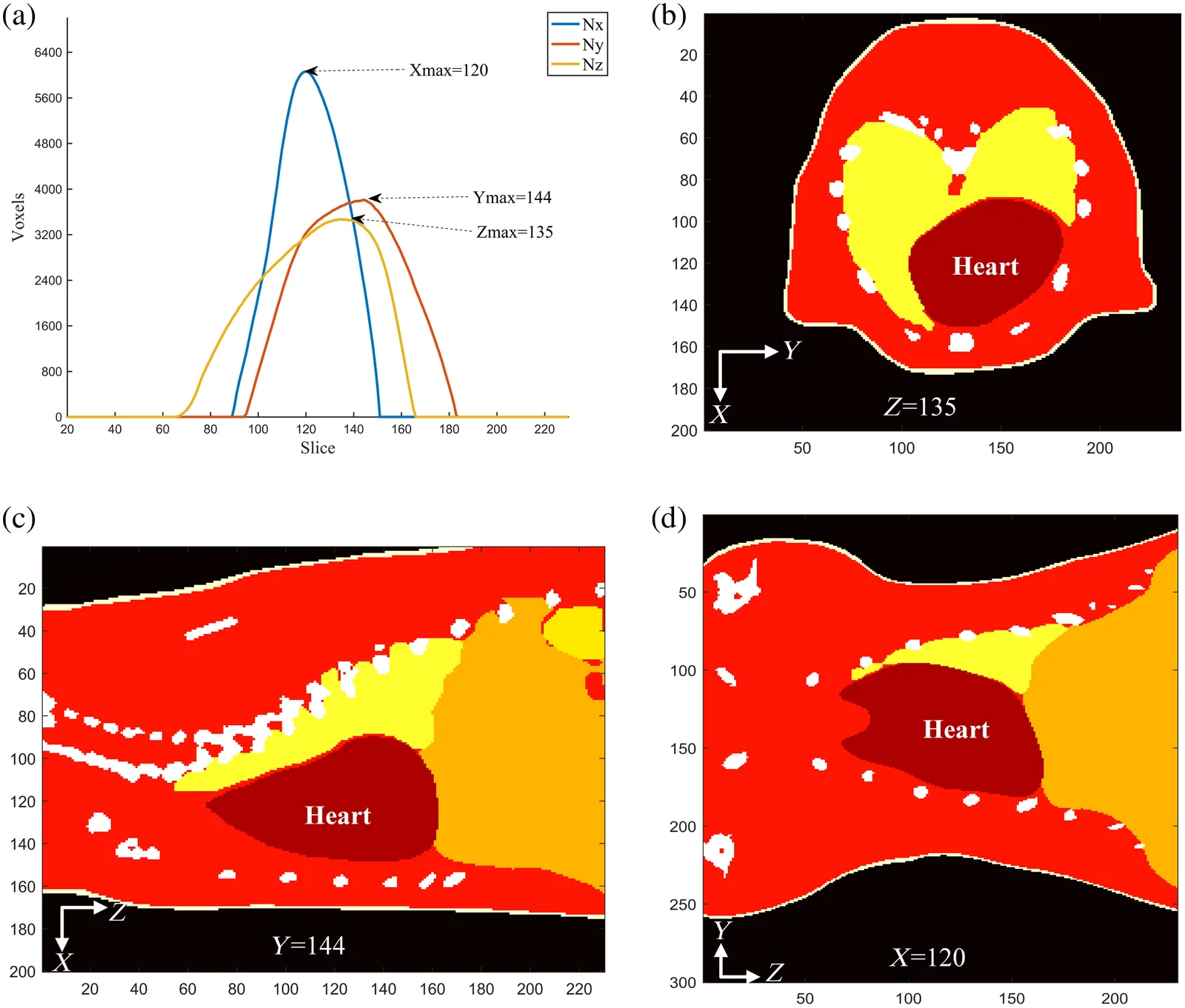
(a) The heart voxels on every slice of the X, Y, and Z-axis. (b) The 135th slice has the most heart voxels on the Z-axis. (c) The 144th slice has the most heart voxels on the Y-axis. (D) The 120th slice has the most heart voxels on the X-axis.

Because the heart is located deep within the thoracic cavity, diffused photons must traverse complex tissue types and structures. Identifying an optimal light source position is therefore essential to maximize photon delivery to the heart. We selected a 7×14 grid of virtual point light sources ($A_{i}$, $B_{i}$, $C_{i}$, $D_{i}$, $E_{i}$, $F_{i}$, $G_{i}$, i=1,2,…,14) as incident points on the thorax skin, as shown in Fig. 3(a). The source is equivalent to a narrow pencil beam with arbitrary directional calibration. Using the heart center [120, 144, 135] as the endpoint, the vector $A_{i}O$ (i=1, 2, 3…14) represents a primary column of incident light, as shown in Fig. 3(b). These positions $B_{i}$, $C_{i}$, $D_{i}$, $E_{i}$, $F_{i}$, and $G_{i}$ were defined on the thorax skin at varying angles relative to $A_{i}O$ – specifically, 5 deg, 10 deg, 15 deg, 20 deg, 25 deg, and 30 deg, respectively. The cosine of the angle between $A_{i}O$ and $B_{i}O$ is expressed as follows: $\;\cos\;\theta =\frac{\mathrm{AiO}\times \mathrm{XiO}}{\left|\mathrm{AiO}\right|\times \left|\mathrm{XiO}\right|}$ , where XiO is a set of vectors $B_{i}O$, $C_{i}O$, $D_{i}O$, $E_{i}O$, $F_{i}O$, and $G_{i}O$. These incident points are separated by 0.1 or 0.05 mm apart along the Z direction. The position coordinates of virtual light sources and the cosine value of the incident direction were presented in the Supplementary Material, Table S1.

**Fig. 3:**
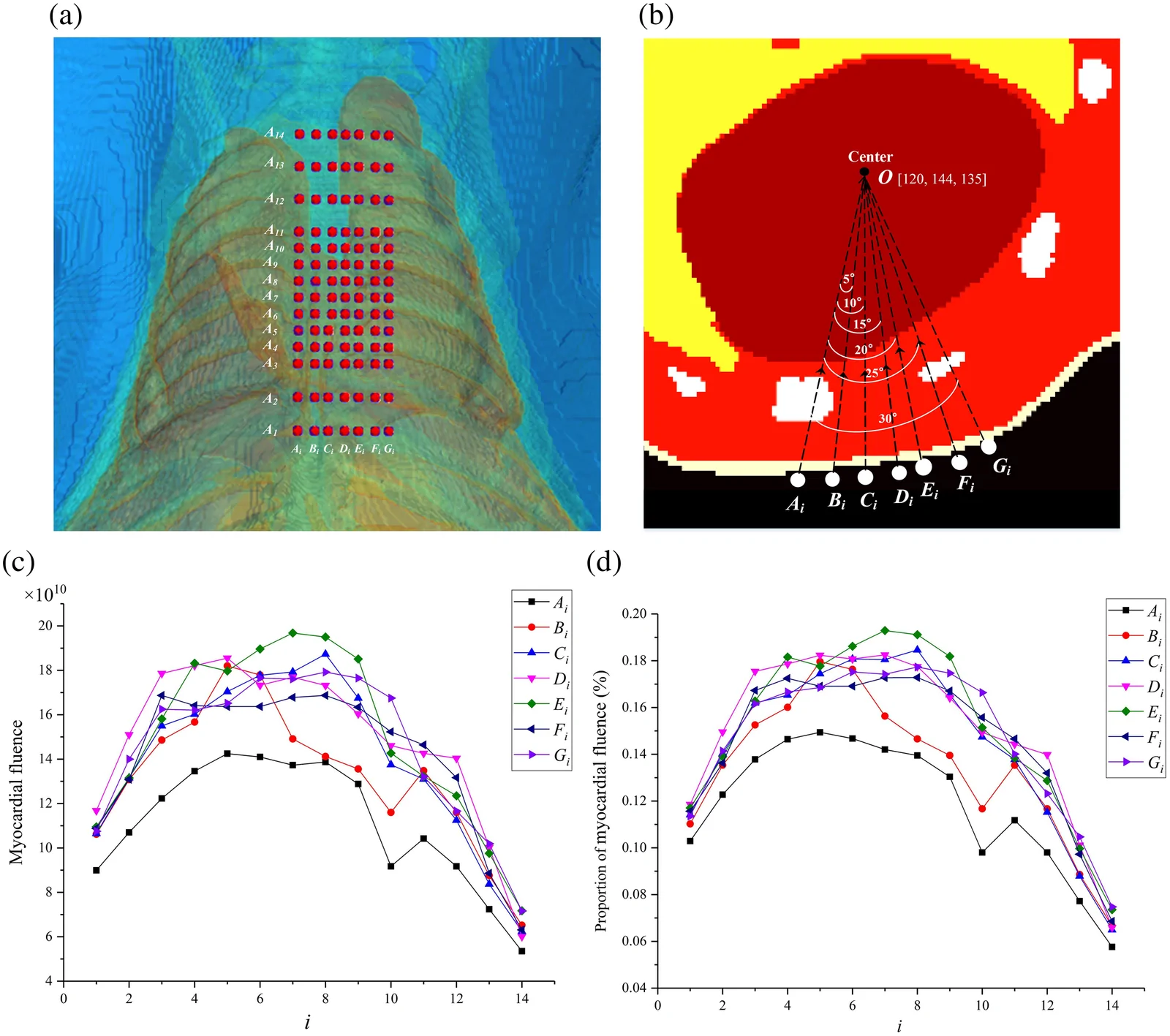
(a) 7×14 virtual light sources $A_{i}$, $B_{i}$, $C_{i}$, $D_{i}$, $E_{i}$, $F_{i}$, $G_{i}$ (i=1, 2, …, 14), were distributed on the 3D mouse body. Fourteen virtual light sources ($A_{1},A_{2},\ldots ,A_{14}$) on the front of the sternum are separated by 0.1 or 0.05 mm in the Z direction. (b) The vectors of incident light are $A_{i}O$, $B_{i}O$, $C_{i}O$, $D_{i}O$, $E_{i}O$, $F_{i}O$, and $G_{i}O$. These light sources are projected into the heart center O [120, 144, 135] at adjacent 5 deg intervals. The coordinates of $B_{i}$, $C_{i}$, $D_{i}$, $E_{i}$, $F_{i}$, and $G_{i}$ are calculated their coordinates are calculated based on the law of cosine for vectors and skin-edge conditions. (c) The myocardial fluence from 7×14 virtual light sources $A_{i}$, $B_{i}$, $C_{i}$, $D_{i}$, $E_{i}$, $F_{i}$, $G_{i}$ (i=1, 2, 3, …, 14). The fluence on the myocardium from the $E_{7}$ light source is the highest. (d) Percentage of the myocardium fluence for seven types of tissues. The proportion from the $E_{7}$ light source is the highest.

### 3.2 Optimal Light Source Location

Having established the parameters for the 3D optical diffusion simulation (including the tissue model, light source positions, incident directions, and optical properties listed in Table 1), we evaluated the simulation outcomes. Figures 3(c) and 3(d) illustrate the light fluence and photon absorption within the thoracic cavity derived from the MC simulation. The $E_{7}$ light source, located at [170, 147, 130], yielded both the highest myocardial light fluence and the greatest number of photons absorbed by the myocardium. Consequently, [170, 147, 130] was identified as the optimal incident light source position. Anatomically, this optimal source is located between the sixth and seventh ribs on the left side of the chest, at a horizontal distance of ∼1.8  mm from the sternum.

### 3.3 Optimal Light Fluence Distribution and Photon Absorption

The light fluence and photon absorption are the weights of all photon traversal and absorption events within each tissue voxel. The distributions were calculated across all 208×308×992  voxels, categorized by every tissue type. Figure 4 illustrates the light fluence distribution and photon absorption within the thoracic cavity using the optimal light source configuration. Figures 4(a) and 4(b) display the myocardial light fluence distribution in the coronal and horizontal planes, respectively, with photons injected from the right side of the sternum between the 6th and 7th ribs. The results confirm that effective photons (light fluence ≥1) can penetrate to the center of the myocardium, although they do not uniformly illuminate the entire myocardial region. The pie charts in Figs. 4(c) and 4(d) quantify the contributions of different tissues to overall light fluence and absorption. Under the optimized source position and orientation, the myocardium accounted for 9.5% of the total light fluence and absorbed 24.9% of all injected photons. Notably, nearly half of the propagating photons were absorbed by muscle tissue during transmission through the thoracic wall.

**Fig. 4:**
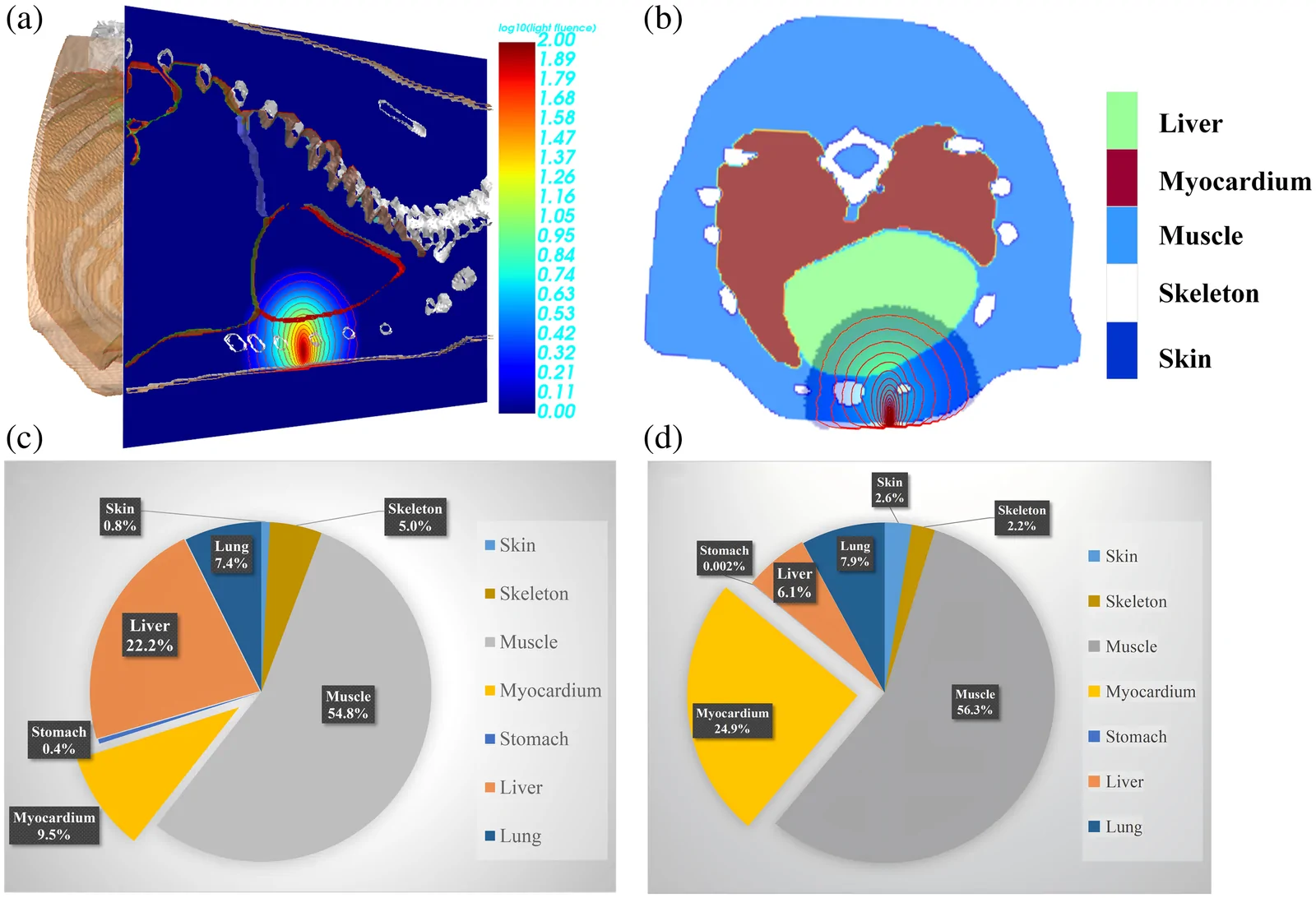
(a) 3D rendering of light fluence distribution generated by an optimal light source with incident voxel position [170, 147, 130] and direction (−0.9933, −0.0596, 0.0993) on the X–Z plane at Y=147. (b) Light fluence distribution on the X–Y plane at Z=130. (c) Pie chart of light fluence across seven thoracic tissues. (d) Pie chart of light absorption by seven thoracic tissues.

### 3.4 Optimal Detector Location

After determining the optimal position and direction for the light source, it is necessary to identify the most effective detector position for diffuse optical imaging. Utilizing the optimal source coordinates [170, 147, 130] in the simulation model, we calculated the DPF, PPF, and the PPF/DPF ratio to evaluate the feasibility of detecting signals from the target tissue. Centered around the light source, virtual detectors were placed in four directions (horizontal and vertical) across the mouse thorax, with a detection photon capture range defined by a sphere with a 1.5 mm radius, as shown in Fig. 5(a). The size of the virtual detector refers to the photodetector (OPT101, Texas Instruments, Dallas, Texas, United States) that we used before.^11^ The position coordinates of virtual detectors in four directions are presented in the Supplementary Material, Table S2.

**Fig. 5:**
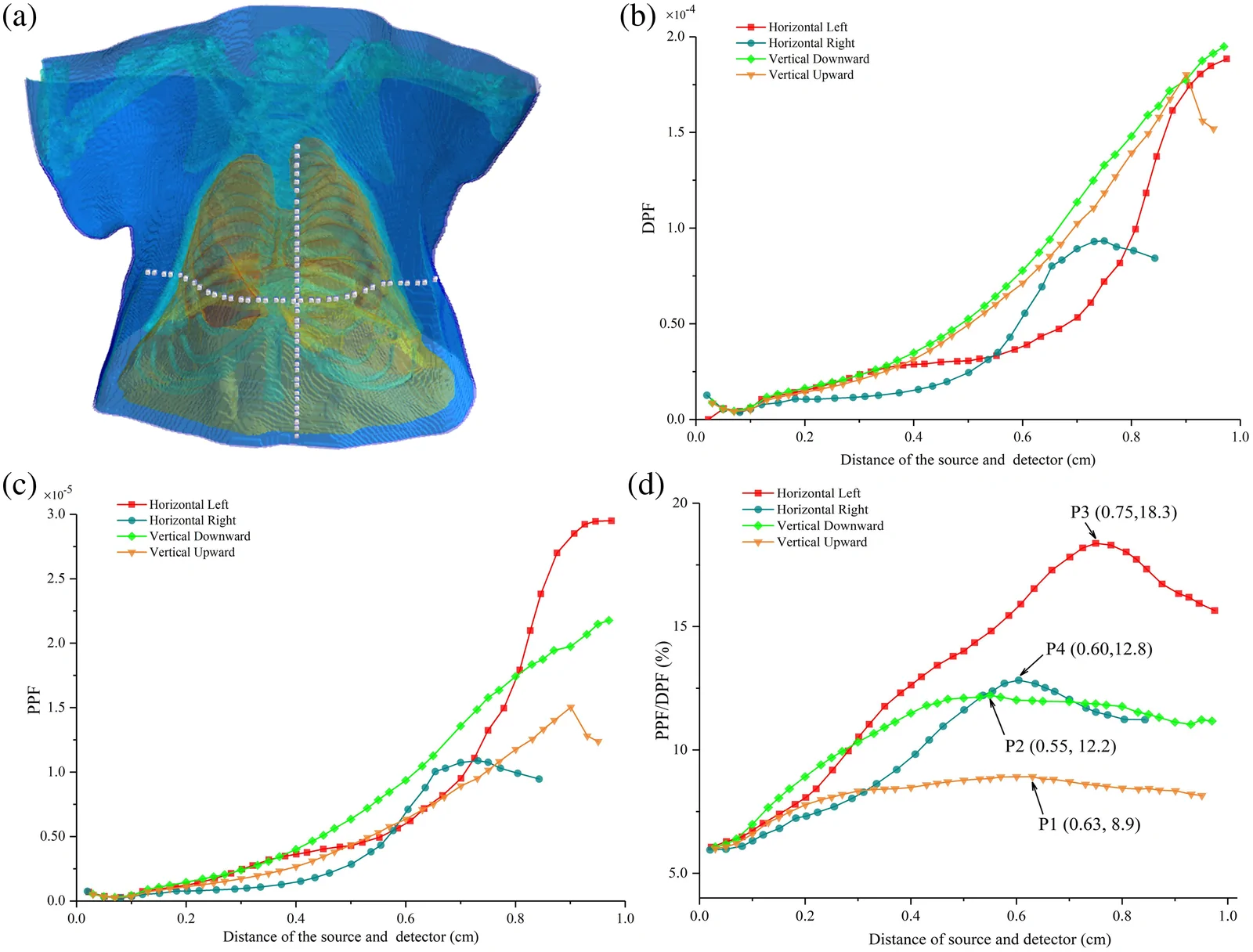
(a) Virtual sphere photodetectors distributed on a 3D mouse body at one-sphere intervals along four directions of the optimal light source: horizontal left direction, horizontal right direction, vertical downward direction, and vertical upward direction. (b)–(d) DPF, PPF, and PPF/DPF change with the distance between the light source and detector, respectively.

The DPF, PPF, and DPF/PPF ratio were calculated based on the simulated photon detection. The variations of these metrics relative to the source-detector distance ($L_{S\text{-}D}$) are plotted in Figs. 5(b)–5(d). During the initial increasing phase, the trends for DPF and PPF resemble those observed in brain simulations,^33^ increasing exponentially as $L_{S\text{-}D}$ grows. However, these trends differ between the horizontal and vertical axes. The highest PPF and PPF/DPF values were observed in the horizontal left direction. As shown in Fig. 5(d), the PPF/DPF ratio gradually increases to a peak before decreasing. We designated the location of this maximum PPF/DPF ratio as the optimal photon-detection site, where photodetectors can capture the highest proportion of myocardial photons. The optimal detection positions were identified as $P_{3}$ [149, 75, 130], $P_{4}$ [145, 202, 130], $P_{2}$ [170, 147, 75], and $P_{1}$ [172, 147, 190] for the horizontal left, horizontal right, vertical downward, and vertical upward directions, respectively.

### 3.5 Photon Propagation in the Ischemic Myocardium by MC Simulation

To further establish an optical diffusion path capable of detecting cardiac oxygenation disorders, we simulated myocardial infarction by altering the optical properties of the myocardium. In Fig. 6(a), we simulated photon propagation with an optimal light source and four detectors at $P_{1}$ [172, 147, 190], $P_{2}$ [170, 147, 75], $P_{3}$ [149, 75, 130], and $P_{4}$ [145, 202, 130]. The myocardial light fluence and the number of detected photons are related to the scattering coefficient $\mu_{s}/\mu_{s0}$ in Figs. 6(b)–6(c). Here, $\mu_{s0}$ is the normal scattering coefficient, and $\mu_{s}$ is taken as 50%, 55%, 60%, 65%, 70%, 75%, 80%, 85%, 90%, 95%, and 100% of the normal scattering coefficient, respectively.

**Fig. 6:**
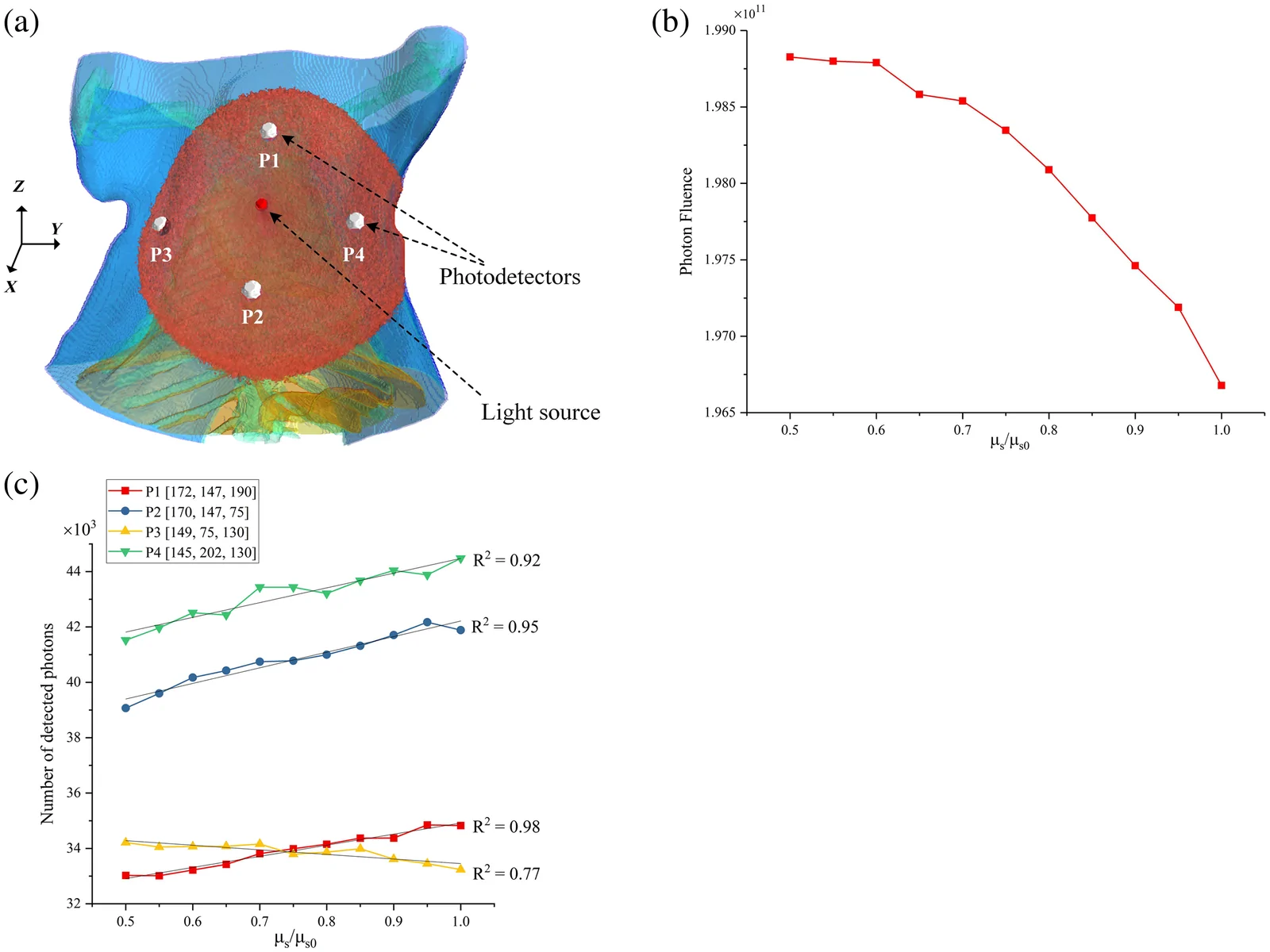
(a) Optimal light source located at [170, 147, 130] and four optimal detectors located at $P_{1}$ [172, 147, 190], $P_{2}$ [170, 147, 75], $P_{3}$ [149, 75, 130], and $P_{4}$ [145, 202, 130]. (b) The myocardial light fluence changes with the scattering coefficient $\mu_{s}/\mu_{s0}$. (c) The number of the detected photons changed with $\mu_{s}/\mu_{s0}$ at $P_{1}$, $P_{2}$, $P_{3}$, and $P_{4}$ sites.

As shown in Fig. 6(b), the scattering coefficient decreases, and the scattering ratio $\mu_{s}/\mu_{s0}$ gradually declines, indicating progressive myocardial ischemia. The myocardial light fluence shows an increasing trend, conversely. This may be because the light path and penetration depth of total photons increase, resulting in increased light fluence in the myocardial region. As illustrated in Fig. 6(c), the number of detected photons at the four virtual spherical detectors ($P_{1}$, $P_{2}$, $P_{3}$, and $P_{4}$) placed on the body surface exhibits distinct responses to the ischemia severity index $\mu_{s}/\mu_{s0}$. At the $P_{1}$, $P_{2}$, and $P_{4}$ sites, the number of detected photons consistently decreases as the ischemia worsens. Due to the variations in the anatomical paths traversed during photon diffusion, there are differences in the absolute number of detected photons at these three sites. Linear regression analysis reveals that the variations in photon counts at these three sites exhibit excellent linear correlations with the severity of ischemia (adjusted $R^{2}>0.9$). However, at the $P_{3}$ site, the number of detected photons displayed an anomalous increasing trend as ischemia exacerbates, resulting in a relatively poor linear goodness-of-fit (adjusted $R^{2}=0.72$). This discrepancy is primarily attributed to anatomical constraints: the heart is situated toward the left side of the thoracic cavity and is partially surrounded by the lungs. During detection at position $P_{3}$, the photon path traverses the right lung tissue, suffering from severe absorption and scattering interference. Therefore, comprehensively considering the stability and correlation of the detected light intensity, $P_{4}$ emerges as the optimal detection site for body surface monitoring.

## 4 Discussion

In this study, we employed Monte Carlo simulation of photon propagation in mouse cardiothoracic tissues to characterize optical diffusion in the mouse heart and demonstrated the theoretical feasibility of optical detection for evaluating myocardial ischemia. Using the Digimouse atlas, a 3D voxel model of the mouse cardiothoracic tissues was constructed. The light source position that maximizes light fluence in the heart was identified, along with the optimal detection location for myocardial signals. The photon propagation characteristics at different positions revealed a significant influence of tissue structure on light diffusion paths, which provides valuable guidance for optimizing the configuration of optical imaging. Furthermore, we observed that at a wavelength of 800 nm, the optical signals detected on the body surface at certain locations exhibited a linear correlation with the severity of ischemia. A reasonable layout of light sources and detectors is essential for acquiring high - quality optical signals. These findings provide an optimized S-D configuration for continuous-wave, time-domain, and frequency-domain NIRS in cardiac function assessment.

### 4.1 Cardiac Diffuse Optical Effective Depth

In recent years, several studies have explored diffuse optical simulations of cardiac tissue.^34^^,^^35^ Although the photon diffusion patterns in the cardiac region reported in these previous studies are consistent with our findings, their simulations were often restricted to shallow millimeter-scale spatial dimensions because of the use of overly simplified tissue models. Furthermore, most of these studies focused exclusively on *ex vivo* hearts.^35^ In contrast, the present study constructed a highly realistic and complex anatomical model based on the Digimouse dataset. Although the mouse heart is located at a depth of approximately 10 mm, our simulation results confirm that photons can effectively penetrate from the skin surface and diffuse into the deep myocardial tissue, achieving a penetration depth of up to 15 mm. For comparison, in the highly mature field of functional near-infrared spectroscopy (fNIRS) for brain imaging, the current typical depth sensitivity of most continuous-wave-based imaging is also approximately 15 mm.^36^ Therefore, we deduce that the noninvasive NIRS probing of the mouse heart in vivo is entirely feasible from the perspective of physical optical transport mechanisms.

### 4.2 Myocardial Optical Absorption

Compared with fNIRS brain imaging—where photons traverse the scalp, skull, and cerebrospinal fluid—cardiac diffuse optical imaging involves a far more complex photon migration path through highly heterogeneous tissues, including skin, adipose tissue, skeletal muscle, and the lungs. As shown in Fig. 4(d), muscle tissue accounts for 54.6% of photon absorption during cardiothoracic diffusion in our model. While this seemingly contrasts with human thoracic simulations (which report ∼12% absorption in skeletal muscle and ∼47% in fat),^10^ the discrepancy is merely an artifact of the Digimouse atlas anatomical simplification. By merging subcutaneous fat and skeletal muscle into a single “muscle” layer [Layer 3, shown in Fig. 1(c)], this combined structure naturally exhibits the highest optical absorption and photon fluence. Crucially, the combined absorption rate of muscle and fat in the human model (59%) is in excellent agreement with our resultant 54.6%. This consistency effectively resolves the apparent data discrepancy and confirms the reliability of our simplified model in replicating equivalent cardiothoracic light transport.

### 4.3 Hemodynamics from the Skeletal Muscle or Myocardium

Skeletal muscle is a major photon absorber in diffuse optics, with attenuation driven largely driven by hemoglobin, myoglobin, and cytochrome c oxidase.^36^^,^^37^ Thus, a significant portion of migrating photons in the cardiothoracic region is absorbed by the chest wall. Crucially, however, this superficial absorption does not practically interfere with the detection of myocardial ischemia due to distinct hemodynamic profiles. The chest wall and the myocardium possess independent vascular networks—supplied by the systemic circulation and coronary arteries, respectively. Consequently, localized coronary occlusions that trigger myocardial ischemia do not perturb the stable oxygenation of the overlying skeletal muscle. This decoupling is supported by prior in vivo rat models, which demonstrated stable local oxygen levels in skeletal muscle during induced myocardial ischemia.^11^ Therefore, we deduce that the NIRS or DOI signal variations observed during ischemic events are fundamentally derived from the deep myocardium, free from superficial muscle confounding.

### 4.4 Optical Absorption and Scattering Parameters in Myocardial Ischemia

Our simulation model strategically utilizes the near-infrared isosbestic point of HbR and ${\mathrm{HbO}}_{2}$ (797 to 805 nm).^30^^,^^38^ Specifically, we assumed that at 800 nm, myocardial ischemia alters only the scattering coefficient, while leaving the absorption coefficient unchanged. This assumption is justified because absorption at 800 nm depends strictly on total hemoglobin concentration (HbT)—which remains approximately stable during acute or isovolumic ischemia—and is entirely unaffected by declining oxygen saturation. Conversely, ischemia triggers micro-morphological changes in cardiomyocytes that significantly reduce tissue scattering.^15^ By isolating these variables and manipulating solely the scattering coefficient to simulate ischemia, our model robustly demonstrates the specific sensitivity of photon migration paths to ischemia-induced structural abnormalities in cardiac tissue.

### 4.5 Limitations

This study determined the optimal configurations for light sources and detectors by using the MCVM photon propagation framework. This simulation-guided approach not only enhances the spatial consistency of optical monitoring but also reduces the empirical trial-and-error time for S-D placement during animal experiments. Moving forward, integrating other robust MC frameworks (e.g., MCX^39^^,^^40^ or MCML^12^) along with higher-resolution digital mouse models could further refine photon path accuracy and assist in designing cardiac multioptode systems. Acknowledging that the current model evaluates only a single S-D pair, our future work will concentrate on multisource and multichannel simulations. These efforts aim to establish a comprehensive theoretical framework for diffuse optical tomography in cardiac hemodynamic monitoring.

## 5 Conclusion

This computational study demonstrates the feasibility of diffuse optical techniques for the transcutaneous detection of the mouse myocardial ischemia. By quantifying photon migration, we have demonstrated that an optimized source-detector (S-D) configuration effectively isolates myocardial-specific signals, establishing a linear correlation between optical signal variations and ischemic severity. As the first comprehensive theoretical investigation of diffuse optics in the mouse cardiac applications, this work addresses the critical limitations of current non-invasive monitoring approaches. The theoretical framework represents a significant advancement toward the clinical translation of near-infrared spectroscopy and diffuse optical imaging for the real-time *in vivo* assessment of cardiac hemodynamics and tissue oxygenation in murine models.

## Supplementary Material

10.1117/1.JBO.31.10.105001.s01

10.1117/1.JBO.31.10.105001.s02

## Data Availability

The code and data that support the findings during the current study are available from the corresponding author upon reasonable request.
