# Supplementary material for: Spatial optimization of transthoracic diffuse optics for myocardial ischemia in mice: a Monte Carlo study
Source: J Biomed Opt. 2026 Oct 14;31(10):105001. doi: 10.1117/1.JBO.31.10.105001 (PMC13667762; doi:10.1117/1.JBO.31.10.105001)
Supplement: Supplementary file 1 [file JBO_031_105001_SD001.docx]

Table S1 Virtual light source coordinates and cosine of incident direction for angles 0° to 30° with 5° intervals.

| **poisition** | **angle** | $\boldsymbol{x}$ | $\boldsymbol{y}$ | $\boldsymbol{z}$ | $\boldsymbol{\alpha}$ | $\boldsymbol{\beta}$ | $\boldsymbol{\gamma}$ |
| --- | --- | --- | --- | --- | --- | --- | --- |
| A_1_ | 0° | 172 | 129 | 85 | -0.7076 | 0.1905 | 0.6804 |
| A_2_ | 0° | 172 | 129 | 95 | -0.7691 | 0.2111 | 0.6032 |
| A_3_ | 0° | 172 | 129 | 105 | -0.8388 | 0.2303 | 0.4934 |
| A_4_ | 0° | 172 | 129 | 115 | -0.9052 | 0.2437 | 0.3482 |
| A_5_ | 0° | 172 | 129 | 120 | -0.9302 | 0.2504 | 0.2683 |
| A_6_ | 0° | 172 | 129 | 125 | -0.9494 | 0.2556 | 0.1826 |
| A_7_ | 0° | 172 | 129 | 130 | -0.9615 | 0.2589 | 0.0925 |
| A_8_ | 0° | 172 | 129 | 135 | -0.9656 | 0.2600 | 0.0000 |
| A_9_ | 0° | 172 | 129 | 140 | -0.9615 | 0.2589 | -0.0925 |
| A_10_ | 0° | 172 | 129 | 145 | -0.9494 | 0.2556 | -0.1826 |
| A_11_ | 0° | 173 | 129 | 150 | -0.9342 | 0.2336 | -0.2695 |
| A_12_ | 0° | 173 | 129 | 155 | -0.9124 | 0.2106 | -0.3509 |
| A_13_ | 0° | 173 | 129 | 165 | -0.8520 | 0.1802 | -0.4915 |
| A_14_ | 0° | 173 | 129 | 175 | -0.7836 | 0.1507 | -0.6027 |
| B_1_ | 5° | 171 | 134 | 85 | -0.7072 | 0.1387 | 0.6933 |
| B_2_ | 5° | 171 | 134 | 95 | -0.7777 | 0.1525 | 0.6099 |
| B_3_ | 5° | 171 | 134 | 105 | -0.8499 | 0.1666 | 0.4999 |
| B_4_ | 5° | 171 | 134 | 115 | -0.9158 | 0.1796 | 0.3592 |
| B_5_ | 5° | 171 | 134 | 120 | -0.9428 | 0.1849 | 0.2773 |
| B_6_ | 5° | 171 | 134 | 125 | -0.9636 | 0.1889 | 0.1889 |
| B_7_ | 5° | 171 | 134 | 130 | -0.9768 | 0.1915 | 0.0958 |
| B_8_ | 5° | 172 | 134 | 135 | -0.9820 | 0.1888 | 0.0000 |
| B_9_ | 5° | 172 | 134 | 140 | -0.9777 | 0.1880 | -0.0940 |
| B_10_ | 5° | 172 | 134 | 145 | -0.9650 | 0.1856 | -0.1856 |
| B_11_ | 5° | 172 | 134 | 150 | -0.9448 | 0.1817 | -0.2725 |
| B_12_ | 5° | 172 | 134 | 155 | -0.9187 | 0.1767 | -0.3533 |
| B_13_ | 5° | 172 | 134 | 165 | -0.8544 | 0.1643 | -0.4929 |
| B_14_ | 5° | 172 | 134 | 175 | -0.7836 | 0.1507 | -0.6027 |
| C_1_ | 10° | 171 | 139 | 85 | -0.7123 | 0.0698 | 0.6984 |
| C_2_ | 10° | 171 | 139 | 95 | -0.7845 | 0.0769 | 0.6153 |
| C_3_ | 10° | 171 | 139 | 105 | -0.8589 | 0.0842 | 0.5052 |
| C_4_ | 10° | 171 | 139 | 115 | -0.9271 | 0.0909 | 0.3636 |
| C_5_ | 10° | 171 | 139 | 120 | -0.9551 | 0.0936 | 0.2809 |
| C_6_ | 10° | 171 | 139 | 125 | -0.9768 | 0.0958 | 0.1915 |
| C_7_ | 10° | 171 | 139 | 130 | -0.9905 | 0.0971 | 0.0971 |
| C_8_ | 10° | 171 | 139 | 135 | -0.9952 | 0.0976 | 0.0000 |
| C_9_ | 10° | 171 | 139 | 140 | -0.9905 | 0.0971 | -0.0971 |
| C_10_ | 10° | 172 | 138 | 145 | -0.9758 | 0.1126 | -0.1876 |
| C_11_ | 10° | 172 | 138 | 150 | -0.9550 | 0.1102 | -0.2755 |
| C_12_ | 10° | 172 | 138 | 155 | -0.9280 | 0.1071 | -0.3569 |
| C_13_ | 10° | 172 | 138 | 165 | -0.8619 | 0.0994 | -0.4972 |
| C_14_ | 10° | 172 | 138 | 175 | -0.7893 | 0.0911 | -0.6072 |
| D_1_ | 15° | 170 | 143 | 85 | -0.7070 | 0.0141 | 0.7070 |
| D_2_ | 15° | 170 | 143 | 95 | -0.7808 | 0.0156 | 0.6246 |
| D_3_ | 15° | 170 | 143 | 105 | -0.8574 | 0.0171 | 0.5144 |
| D_4_ | 15° | 170 | 143 | 115 | -0.9283 | 0.0186 | 0.3713 |
| D_5_ | 15° | 171 | 143 | 120 | -0.9592 | 0.0188 | 0.2821 |
| D_6_ | 15° | 171 | 143 | 125 | -0.9811 | 0.0192 | 0.1924 |
| D_7_ | 15° | 171 | 143 | 130 | -0.9950 | 0.0195 | 0.0976 |
| D_8_ | 15° | 171 | 143 | 135 | -0.9998 | 0.0196 | 0.0000 |
| D_9_ | 15° | 171 | 143 | 140 | -0.9950 | 0.0195 | -0.0976 |
| D_10_ | 15° | 171 | 143 | 145 | -0.9811 | 0.0192 | -0.1924 |
| D_11_ | 15° | 171 | 143 | 150 | -0.9592 | 0.0188 | -0.2821 |
| D_12_ | 15° | 171 | 143 | 155 | -0.9308 | 0.0183 | -0.3650 |
| D_13_ | 15° | 172 | 143 | 165 | -0.8661 | 0.0167 | -0.4997 |
| D_14_ | 15° | 170 | 143 | 85 | -0.7070 | 0.0141 | 0.7070 |
| E_1_ | 20° | 170 | 147 | 85 | -0.7065 | -0.0424 | 0.7065 |
| E_2_ | 20° | 170 | 147 | 95 | -0.7800 | -0.0468 | 0.6240 |
| E_3_ | 20° | 170 | 147 | 105 | -0.8564 | -0.0514 | 0.5138 |
| E_4_ | 20° | 170 | 147 | 115 | -0.9270 | -0.0556 | 0.3708 |
| E_5_ | 20° | 170 | 147 | 120 | -0.9562 | -0.0574 | 0.2869 |
| E_6_ | 20° | 170 | 147 | 125 | -0.9789 | -0.0587 | 0.1958 |
| E_7_ | 20° | 170 | 147 | 130 | -0.9933 | -0.0596 | 0.0993 |
| E_8_ | 20° | 170 | 147 | 135 | -0.9982 | -0.0599 | 0.0000 |
| E_9_ | 20° | 170 | 147 | 140 | -0.9933 | -0.0596 | -0.0993 |
| E_10_ | 20° | 171 | 147 | 145 | -0.9797 | -0.0576 | -0.1921 |
| E_11_ | 20° | 171 | 147 | 150 | -0.9578 | -0.0563 | -0.2817 |
| E_12_ | 20° | 171 | 147 | 155 | -0.9296 | -0.0547 | -0.3645 |
| E_13_ | 20° | 171 | 147 | 165 | -0.8608 | -0.0506 | -0.5064 |
| E_14_ | 20° | 171 | 147 | 175 | -0.7860 | -0.0462 | -0.6165 |
| F_1_ | 25° | 169 | 152 | 85 | -0.6954 | -0.1135 | 0.7096 |
| F_2_ | 25° | 169 | 152 | 95 | -0.7685 | -0.1255 | 0.6274 |
| F_3_ | 25° | 169 | 152 | 105 | -0.8447 | -0.1379 | 0.5172 |
| F_4_ | 25° | 170 | 152 | 115 | -0.9184 | -0.1469 | 0.3674 |
| F_5_ | 25° | 170 | 152 | 120 | -0.9468 | -0.1515 | 0.2840 |
| F_6_ | 25° | 170 | 152 | 125 | -0.9687 | -0.1550 | 0.1937 |
| F_7_ | 25° | 170 | 152 | 130 | -0.9827 | -0.1572 | 0.0983 |
| F_8_ | 25° | 170 | 152 | 135 | -0.9874 | -0.1580 | 0.0000 |
| F_9_ | 25° | 170 | 152 | 140 | -0.9827 | -0.1572 | -0.0983 |
| F_10_ | 25° | 170 | 152 | 145 | -0.9687 | -0.1550 | -0.1937 |
| F_11_ | 25° | 170 | 152 | 150 | -0.9468 | -0.1515 | -0.2840 |
| F_12_ | 25° | 170 | 152 | 155 | -0.9184 | -0.1469 | -0.3674 |
| F_13_ | 25° | 171 | 152 | 165 | -0.8542 | -0.1340 | -0.5024 |
| F_14_ | 25° | 171 | 152 | 175 | -0.7809 | -0.1225 | -0.6125 |
| G_1_ | 30° | 168 | 156 | 85 | -0.6824 | -0.1706 | 0.7108 |
| G_2_ | 30° | 168 | 156 | 95 | -0.7544 | -0.1886 | 0.6287 |
| G_3_ | 30° | 168 | 156 | 105 | -0.8296 | -0.2074 | 0.5185 |
| G_4_ | 30° | 169 | 156 | 115 | -0.9029 | -0.2211 | 0.3685 |
| G_5_ | 30° | 169 | 156 | 120 | -0.9310 | -0.2280 | 0.2850 |
| G_6_ | 30° | 169 | 156 | 125 | -0.9528 | -0.2333 | 0.1944 |
| G_7_ | 30° | 169 | 156 | 130 | -0.9666 | -0.2367 | 0.0986 |
| G_8_ | 30° | 169 | 156 | 135 | -0.9713 | -0.2379 | 0.0000 |
| G_9_ | 30° | 169 | 156 | 140 | -0.9666 | -0.2367 | -0.0986 |
| G_10_ | 30° | 169 | 156 | 145 | -0.9528 | -0.2333 | -0.1944 |
| G_11_ | 30° | 170 | 156 | 150 | -0.9335 | -0.2240 | -0.2800 |
| G_12_ | 30° | 170 | 156 | 155 | -0.9062 | -0.2175 | -0.3625 |
| G_13_ | 30° | 170 | 156 | 165 | -0.8399 | -0.2016 | -0.5039 |
| G_14_ | 30° | 170 | 156 | 175 | -0.7675 | -0.1842 | -0.6140 |
