# Supplementary material for: Spatial optimization of transthoracic diffuse optics for myocardial ischemia in mice: a Monte Carlo study
Source: J Biomed Opt. 2026 Oct 14;31(10):105001. doi: 10.1117/1.JBO.31.10.105001 (PMC13667762; doi:10.1117/1.JBO.31.10.105001)
Supplement: Supplementary file 2 [file JBO_031_105001_SD002.docx]

Table S2 Coordinates of virtual detectors in the four orthogonal directions (horizontal left, horizontal right, vertical downward, vertical upward) with respect to the optimal light source at incident position [170, 147, 130].

| direction | $\boldsymbol{x}$ | $\boldsymbol{y}$ | $\boldsymbol{z}$ |
| --- | --- | --- | --- |
| horizontal left | 148 | 52 | 130 |
| horizontal left | 148 | 55 | 130 |
| horizontal left | 148 | 57 | 130 |
| horizontal left | 148 | 59 | 130 |
| horizontal left | 149 | 62 | 130 |
| horizontal left | 149 | 65 | 130 |
| horizontal left | 149 | 67 | 130 |
| horizontal left | 149 | 69 | 130 |
| horizontal left | 149 | 72 | 130 |
| horizontal left | 149 | 75 | 130 |
| horizontal left | 151 | 77 | 130 |
| horizontal left | 153 | 79 | 130 |
| horizontal left | 155 | 82 | 130 |
| horizontal left | 157 | 85 | 130 |
| horizontal left | 160 | 87 | 130 |
| horizontal left | 162 | 89 | 130 |
| horizontal left | 165 | 92 | 130 |
| horizontal left | 167 | 95 | 130 |
| horizontal left | 168 | 97 | 130 |
| horizontal left | 169 | 99 | 130 |
| horizontal left | 170 | 102 | 130 |
| horizontal left | 171 | 105 | 130 |
| horizontal left | 172 | 107 | 130 |
| horizontal left | 172 | 109 | 130 |
| horizontal left | 172 | 112 | 130 |
| horizontal left | 173 | 115 | 130 |
| horizontal left | 173 | 117 | 130 |
| horizontal left | 173 | 119 | 130 |
| horizontal left | 173 | 122 | 130 |
| horizontal left | 172 | 125 | 130 |
| horizontal left | 172 | 127 | 130 |
| horizontal left | 172 | 129 | 130 |
| horizontal left | 172 | 132 | 130 |
| horizontal left | 171 | 135 | 130 |
| horizontal left | 171 | 137 | 130 |
| horizontal left | 171 | 139 | 130 |
| horizontal left | 171 | 142 | 130 |
| horizontal left | 171 | 145 | 130 |
| horizontal left | 170 | 147 | 130 |
| horizontal left | 170 | 147 | 130 |
| horizontal right | 170 | 149 | 130 |
| horizontal right | 170 | 152 | 130 |
| horizontal right | 169 | 155 | 130 |
| horizontal right | 169 | 157 | 130 |
| horizontal right | 169 | 159 | 130 |
| horizontal right | 168 | 162 | 130 |
| horizontal right | 167 | 165 | 130 |
| horizontal right | 167 | 167 | 130 |
| horizontal right | 166 | 169 | 130 |
| horizontal right | 165 | 172 | 130 |
| horizontal right | 163 | 175 | 130 |
| horizontal right | 162 | 177 | 130 |
| horizontal right | 160 | 179 | 130 |
| horizontal right | 157 | 182 | 130 |
| horizontal right | 155 | 185 | 130 |
| horizontal right | 153 | 187 | 130 |
| horizontal right | 151 | 189 | 130 |
| horizontal right | 148 | 192 | 130 |
| horizontal right | 146 | 195 | 130 |
| horizontal right | 146 | 197 | 130 |
| horizontal right | 145 | 199 | 130 |
| horizontal right | 145 | 202 | 130 |
| horizontal right | 144 | 205 | 130 |
| horizontal right | 144 | 207 | 130 |
| horizontal right | 144 | 209 | 130 |
| horizontal right | 144 | 212 | 130 |
| horizontal right | 143 | 215 | 130 |
| horizontal right | 143 | 217 | 130 |
| horizontal right | 142 | 219 | 130 |
| horizontal right | 141 | 222 | 130 |
| horizontal right | 138 | 225 | 130 |
| vertical downward | 168 | 147 | 33 |
| vertical downward | 168 | 147 | 35 |
| vertical downward | 169 | 147 | 37 |
| vertical downward | 169 | 147 | 40 |
| vertical downward | 170 | 147 | 43 |
| vertical downward | 170 | 147 | 45 |
| vertical downward | 170 | 147 | 47 |
| vertical downward | 170 | 147 | 50 |
| vertical downward | 170 | 147 | 53 |
| vertical downward | 170 | 147 | 55 |
| vertical downward | 170 | 147 | 57 |
| vertical downward | 170 | 147 | 60 |
| vertical downward | 170 | 147 | 65 |
| vertical downward | 170 | 147 | 67 |
| vertical downward | 170 | 147 | 70 |
| vertical downward | 170 | 147 | 73 |
| vertical downward | 170 | 147 | 75 |
| vertical downward | 170 | 147 | 77 |
| vertical downward | 170 | 147 | 80 |
| vertical downward | 170 | 147 | 83 |
| vertical downward | 170 | 147 | 85 |
| vertical downward | 170 | 147 | 87 |
| vertical downward | 170 | 147 | 90 |
| vertical downward | 170 | 147 | 93 |
| vertical downward | 170 | 147 | 95 |
| vertical downward | 170 | 147 | 97 |
| vertical downward | 170 | 147 | 100 |
| vertical downward | 170 | 147 | 103 |
| vertical downward | 170 | 147 | 105 |
| vertical downward | 170 | 147 | 107 |
| vertical downward | 170 | 147 | 110 |
| vertical downward | 170 | 147 | 113 |
| vertical downward | 170 | 147 | 115 |
| vertical downward | 170 | 147 | 117 |
| vertical downward | 170 | 147 | 120 |
| vertical downward | 170 | 147 | 123 |
| vertical downward | 170 | 147 | 125 |
| vertical downward | 170 | 147 | 127 |
| vertical downward | 170 | 147 | 130 |
| vertical upward | 170 | 147 | 133 |
| vertical upward | 170 | 147 | 135 |
| vertical upward | 170 | 147 | 137 |
| vertical upward | 170 | 147 | 140 |
| vertical upward | 171 | 147 | 143 |
| vertical upward | 171 | 147 | 145 |
| vertical upward | 171 | 147 | 147 |
| vertical upward | 171 | 147 | 150 |
| vertical upward | 171 | 147 | 153 |
| vertical upward | 171 | 147 | 155 |
| vertical upward | 171 | 147 | 157 |
| vertical upward | 171 | 147 | 160 |
| vertical upward | 171 | 147 | 163 |
| vertical upward | 171 | 147 | 165 |
| vertical upward | 171 | 147 | 167 |
| vertical upward | 171 | 147 | 170 |
| vertical upward | 171 | 147 | 173 |
| vertical upward | 171 | 147 | 175 |
| vertical upward | 171 | 147 | 177 |
| vertical upward | 172 | 147 | 180 |
| vertical upward | 172 | 147 | 183 |
| vertical upward | 172 | 147 | 185 |
| vertical upward | 172 | 147 | 187 |
| vertical upward | 172 | 147 | 190 |
| vertical upward | 172 | 147 | 193 |
| vertical upward | 172 | 147 | 195 |
| vertical upward | 172 | 147 | 197 |
| vertical upward | 173 | 147 | 200 |
| vertical upward | 173 | 147 | 203 |
| vertical upward | 173 | 147 | 205 |
| vertical upward | 173 | 147 | 207 |
| vertical upward | 173 | 147 | 210 |
| vertical upward | 174 | 147 | 213 |
| vertical upward | 174 | 147 | 215 |
| vertical upward | 174 | 147 | 217 |
| vertical upward | 174 | 147 | 220 |
| vertical upward | 174 | 147 | 223 |
| vertical upward | 174 | 147 | 225 |
